# Oral Health and Swallowing Function of Nursing Home Residents

**DOI:** 10.7759/cureus.62600

**Published:** 2024-06-18

**Authors:** Takafumi Yamano, Kensuke Nishi, Shoichi Kimura, Fumitaka Omori, Kaori Wada, Miho Tanaka, Takashi Tsutsumi

**Affiliations:** 1 Section of Otorhinolaryngology, Department of Medicine, Fukuoka Dental College, Fukuoka, JPN; 2 Section of Otolaryngology, Department of Medicine, Fukuoka Dental College, Fukuoka, JPN; 3 Department of Otorhinolaryngology, Fukuoka Dental College Hospital, Fukuoka, JPN; 4 Department of Nursing, Fukuoka Dental College Hospital, Fukuoka, JPN; 5 Department of General Dentistry, Fukuoka Dental College, Fukuoka, JPN

**Keywords:** the oral health assessment tool, video fluorography, nursing home, swallowing function, oral health

## Abstract

Objective: Although a good oral environment helps reduce the risk of pneumonia in the elderly, repeated pneumonia can occur even with frequent oral care. The actual risk of pneumonia during oral intake, the choice of whether oral intake is possible, and the choice of food form are often determined using video fluorography (VF), which can provide detailed information on swallowing function. However, few reports have compared the oral environment and swallowing function, leaving the relationship unclear. We examined the relationship between the oral environment and swallowing function and the characteristics of swallowing function in elderly nursing home residents.

Methods: The subjects were 48 elderly nursing home residents (13 males, 35 females) with a mean age of 89 years who underwent outpatient or inpatient evaluation of their oral environment and swallowing function. There were three groups of residents: those who were evaluated for swallowing as outpatients, those who were hospitalised for pneumonia, and those who were hospitalised for diseases other than pneumonia. The oral environment was assessed by a dentist or dental hygienist using the Oral Health Assessment Tool (OHAT). Swallowing function was assessed by an otorhinolaryngologist using VF.

Results: There was no correlation between OHAT and VF scores in the outpatient group or the group hospitalised for pneumonia, but there was a correlation in the group hospitalised for reasons other than pneumonia.

Conclusion: In facilities with good oral care, the development of pneumonia may be related to factors other than the oral environment and the OHAT may reflect conditions other than swallowing function. The swallowing function of nursing home residents should be evaluated by VF, which allows observation of all stages of swallowing.

## Introduction

While oral care is effective at preventing pneumonia [1-3], there have been few objective evaluations of the relationship between the oral environment and swallowing function. Although a good oral environment reduces the risk of pneumonia in the elderly, repeated pneumonia can still occur with frequent oral care; the oral environment alone is not a risk factor for pneumonia. The actual risk of pneumonia during oral intake, the choice of whether oral intake is possible, and the choice of food form are often determined using video endoscopy (VE) and video fluorography (VF), both of which can provide detailed information on swallowing function [4].

Previously, we reported on the relationship between the oral environment and swallowing function in elderly patients hospitalized for pneumonia [4]. In the present study, we limited our study to elderly nursing home residents and examined the relationship between oral health and the swallowing function and the characteristics of swallowing function in three groups: those whose swallowing was evaluated on an outpatient basis, those who were hospitalized for pneumonia while in the nursing home, and those who were hospitalized for diseases other than pneumonia.

## Materials and methods

This study was conducted at the Fukuoka Dental College Hospital, Fukuoka. The study was approved by the Fukuoka Gakuen Research Ethics Committee (approval number: 314).

**Figure 1:**
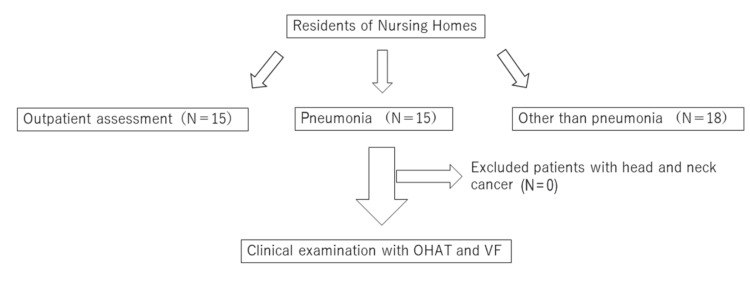
Patient selection flowchart OHAT: Oral Health Assessment Tool; VF: video fluorography

The elderly care facility in this study is affiliated with the Fukuoka Dental College Hospital and oral health care is provided to the residents under the supervision of a full-time dental hygienist after each meal and a weekly visit by a dentist. Residents of the elderly care facility who had their oral environment and swallowing function evaluated at Fukuoka Dental College Hospital between July 2021 and November 2023 were included in the study. Residents who could not give their consent to participate in the study were excluded. Figure 1 shows the flowchart of patient selection.

The following three groups were studied: (i) residents for whom nursing home staff requested an outpatient evaluation of swallowing (n=15), (ii) residents admitted to our hospital from the nursing home for pneumonia (n=15), where pneumonia was diagnosed when individuals had a chest X-ray or chest computed tomography (CT) showing infiltration, with a fever of 37.5°C or higher, an elevated C-reactive protein (CRP) level, a peripheral white blood cell count exceeding 9000/µL, or the presence of any two or more airway symptoms, such as sputum, and (iii) residents admitted to our hospital from the nursing home with issues other than pneumonia (n=18). This latter group included nine cases of acute pyelonephritis, two cases each of gastrointestinal hemorrhage, cerebral infarction, and femur fracture, and one case each of epilepsy, infectious enteritis, and dehydration. There were no head/neck cancers that could have directly affected the oral environment. In all patients, oral intake was maintained and there were no cases of alternative nutrition such as tube feeding. The level of dementia was defined as the ability to perform simple instructional movements.

Evaluation method

The oral environment was assessed by a dentist or dental hygienist using the Oral Health Assessment Tool (OHAT). To equalize oral care so that high-quality oral care is provided regardless of the caregiver, it was ensured that all individuals had undergone periodic oral assessments and followed oral care protocols based on the assessments [5]. The OHAT was developed for the objective assessment of the oral environment in clinical practice, and the items examined are lips, tongue, gingival mucosa, saliva, remaining teeth, dentures, oral cleaning, and toothache, which are each rated on a 3-point scale from 0 to 2. A higher score indicates a poorer oral environment [6].

The swallowing function was evaluated by an otolaryngologist using VF, as described in the earlier study by the authors [4]. Impaired feeding was defined as 0 if 10 cc of contrast medium (Omnipaque 300®; GE HealthCare, Chicago, United States) could be delivered in one full swallow, 1 if less than 50% remained in the oral cavity, and 2 if more than 50% remained. Premature pharyngeal inflow was scored 0 for no and 1 for yes. Laryngeal entry was rated on a 3-point scale (0, no laryngeal entry; 1, laryngeal entry only; 2, aspiration). Elicitation of the swallowing reflex was assessed according to the laryngeal elevation delay time (LEDT), which is considered a useful visual marker during pharyngeal swallowing, with normal defined as a value of 0.35 s or less [7]; prolongation was scored as 0 for no and 1 for yes. Residual in the glottis valley and residual in the pear-shaped depression were rated on a four-point scale. 0 for no residual, 1 for a very small amount, 2 for no overflow into the pharynx, and 3 for overflow into the larynx.

Data analysis

Spearman’s rank correlation coefficient was used to correlate OHAT with the swallowing contrast examination. The Kruskal-Wallis test was used to compare VF scores among the three groups.

## Results

The subjects were 48 nursing home residents (13 males, 35 females) with a mean age of 89 years (range: 70-107 years).

Correlation between OHAT and VF scores

**Figure 2:**
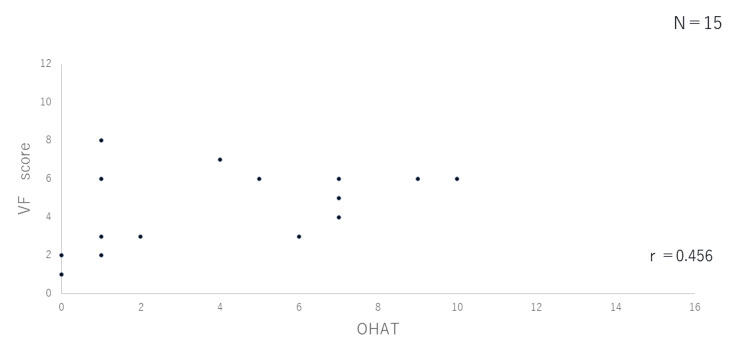
Correlation between OHAT and VF scores (Outpatient group) OHAT: Oral Health Assessment Tool; VF: video fluorography

**Figure 3:**
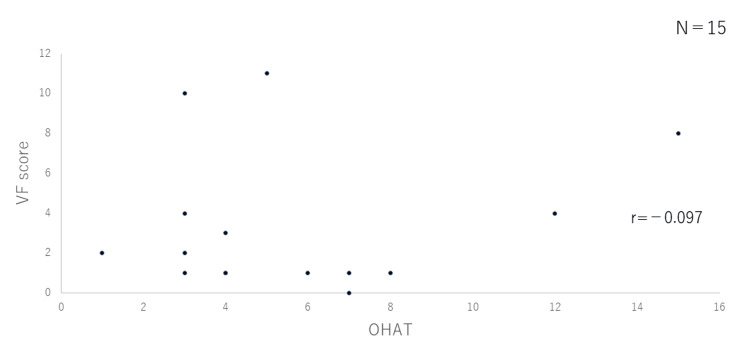
Correlation between OHAT and VF scores (Group hospitalized for pneumonia) OHAT: Oral Health Assessment Tool; VF: video fluorography

**Figure 4:**
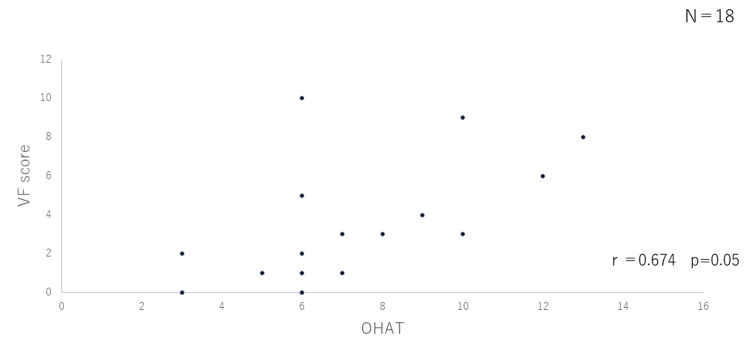
Correlation between OHAT and VF scores (Group hospitalized for issues other than pneumonia) OHAT: Oral Health Assessment Tool; VF: video fluorography

In Figures 2-4, the VF score (vertical axis) is plotted against the OHAT score (horizontal axis) for the three groups. There was no correlation between the two in the outpatient group (r=0.456) or the group hospitalized for pneumonia (r= -0.097), while a correlation was observed in the group hospitalized for issues other than pneumonia (r=0.674; p=0.05).

Distribution of OHAT

**Figure 5:**
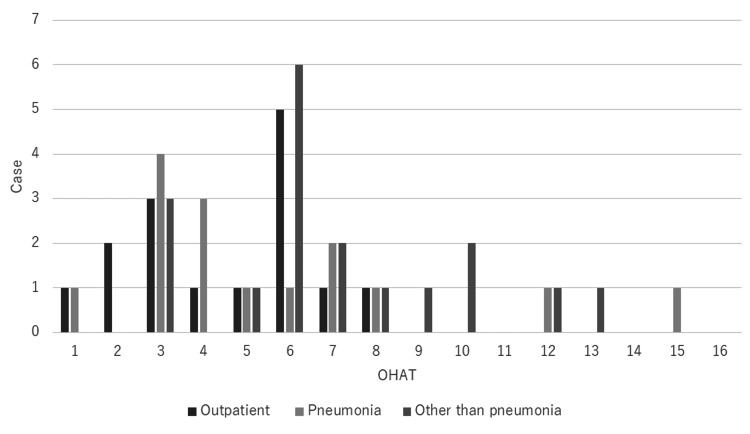
Distribution of OHAT scores OHAT: Oral Health Assessment Tool

In the group examined in the outpatient setting, one, two, three, one, one, five, one, and one patients scored 1 to 8 points, respectively, with no patient scoring 9 or higher. In the group hospitalized for pneumonia, one, four, three, one, one, two, one, one, and one patients scored 1, 3 to 8, 12, and 15 points, respectively. In the group hospitalized for issues other than pneumonia, three, one, six, two, one, one, two, one, and one patients scored 3, 5-10, 12, and 13 points, respectively. More of the group hospitalized for reasons other than pneumonia had higher OHAT scores than the other two groups (Figure 5).

Comparison of VF item scores

The VF scores were compared by item among the three groups: six, eight, and one patients scored 0 to 2, respectively, in the outpatient group versus nine, three, and three patients in the group hospitalized for pneumonia, and nine, nine, and zero patients in the group hospitalized for issues other than pneumonia (Table 1).

**Table 1:** Comparison of VF item scores among the three groups (Feeding disturbance) VF: video fluorography

	Outpatient	Hospitalized for pneumonia	Hospitalized for other than pneumonia
Score 0	6	9	9
Score 1	8	3	9
Score 2	1	3	0

For early pharyngeal influx, the group examined in an outpatient setting had seven cases with a score of 0 and eight cases with a score of 1. The group hospitalized for pneumonia had eight cases with a score of 0 and seven cases with a score of 1. In the group hospitalized for conditions other than pneumonia, 11 patients had a score of 0, and seven patients had a score of 1. The overall oral phase showed 40-60% impairment (Table 2).

**Table 2:** Comparison of VF item scores among the groups (Premature pharyngeal inflow) VF: video fluorography

	Outpatient	Hospitalized for pneumonia	Hospitalized for other than pneumonia
Score 0	7	8	11
Score 1	8	7	7

For laryngeal inflow, the group examined in the outpatient setting had seven, two, and six cases with scores of 0, 1, and 2, respectively, compared to six, three, and six cases in the group hospitalized for pneumonia, and 13, one, and four cases in the group hospitalized for other reasons (Table 3). Laryngeal inflow was somewhat less common in non-pneumonia hospitalizations, while more than 50% of the other two groups showed impairment, although there were no significant differences among the three groups.

**Table 3:** Comparison of VF item scores among the groups (Laryngeal invasion) VF: video fluorography

	Outpatient	Hospitalized for pneumonia	Hospitalized for other than pneumonia
Score 0	7	6	13
Score 1	2	3	1
Score 2	6	6	4

Prolonged LEDT was present in seven cases with a score of 0 and eight cases with a score of 1 in the group examined as outpatients, compared to nine and six cases, respectively, in the group hospitalized for pneumonia, and eight and 10 patients in the group hospitalized for other reasons (Table 4).

**Table 4:** Comparison of VF item scores among the groups (Prolongation of the LEDT) LEDT: laryngeal elevation delay time; VF: video fluorography

	Outpatient	Hospitalized for pneumonia	Hospitalized for other than pneumonia
Score 0	7	9	8
Score 1	8	6	10

For residual in the glottis trough, there were nine, two, three, and one cases with respective scores of 1 to 3 in the group examined as outpatients, nine, five, one, and zero cases in the group hospitalized for pneumonia, and 10, two, five, and zero cases in the group hospitalized for other reasons (Table 5).

**Table 5:** Comparison of VF item scores among the groups (Degree of contrast medium pooling at the vallecula) VF: video fluorography

	Outpatient	Hospitalized for pneumonia	Hospitalized for other than pneumonia
Score 0	9	9	10
Score 1	2	5	2
Score 2	3	1	6
Score 3	1	0	0

For residual in the pear-shaped depression, the group examined in an outpatient setting had 10, one, three, and one cases with scores of 0 to 3, respectively, versus 12, one, zero, and two respective cases in the group hospitalized for pneumonia and 13, two, two, and one cases in the group hospitalized for other reasons (Table 6).

**Table 6:** Comparison of VF item scores among the groups (Degree of contrast medium pooling at the piriform sinuses) VF: video fluorography

	Outpatient	Hospitalized for pneumonia	Hospitalized for other than pneumonia
Score 0	10	12	13
Score 1	1	1	2
Score 2	3	0	2
Score3	1	2	1

The overall pharyngeal phase was 20-55% impaired, although there were no significant differences among the three groups.

## Discussion

There are reports on the effectiveness of oral care in preventing the development of pneumonia due to swallowing in the elderly. In a study by Yanehara et al., the incidence of pneumonia was 11% in elderly care residents who brushed their teeth after each meal and took care of their oral cavity versus 19% in those who did not; the relative risk of pneumonia was 1.7 times higher in the latter group [1]. The reflex threshold decreases significantly after one month of careful oral care in a long-term care facility for the elderly, and the cough reflex threshold decreases after care compared to before the start of care [2]. Oral care by a dental hygienist twice a week for 24 months significantly reduces the frequency of fever and death due to aspiration pneumonia compared to those who did not receive such care [3]. Therefore, elderly care facilities should provide this therapy as part of their treatment for dysphagia. In a previous report on 264 residents at a facility between 2011 and 2015 [8], 37 (14%) developed pneumonia and were transported to the hospital, indicating that thorough oral care in the facility can contribute to the prevention of pneumonia to an extent.

There is no doubt that there is a significant relationship between the oral environment and the onset of pneumonia, but there have been few objective studies of the relationship between the oral environment and swallowing function, which is a determinant of whether oral intake is possible and the choice of food form. To the best of our knowledge, only one study has examined the relationship using a contrast-enhanced swallowing examination [4], which is considered the most reliable method of assessing swallowing function because it can observe the oral, pharyngeal, and oesophageal phases [9] and is extremely helpful in making policy decisions. We always perform a contrast-enhanced swallowing test when deciding on a treatment plan, such as initiating oral feeding after prolonged fasting, swallowing rehabilitation, or surgery to improve swallowing function.

In a study of the relationship between the OHAT score and swallowing function, Nakayama et al. found that the Functional Oral Intake Scale (FOIS), a method of assessing oral intake, was significantly associated with the saliva and denture scores in patients admitted to a convalescent hospital [10]. In comparison, in an analysis of elderly residents of a nursing home that excluded patients with nonoral nutrition, Chou et al. reported no significant association between the FOIS and OHAT scores; the Mini Nutritional Assessment (MNA) did show a correlation [11]. Another report found no association between OHAT, the presence of dysphagic pneumonia, and improved nutritional intake in patients with acute stroke [12]. Previously, the authors of the current study compared OHAT with endoscopic and angiographic swallowing in elderly patients hospitalized for pneumonia and found no correlation [4]. In the present study also, there was no correlation between OHAT and swallowing angiography in patients hospitalised with pneumonia, indicating that factors other than the oral environment are involved in the development of pneumonia in elderly facilities where adequate oral care is provided.

Individuals with dysphagia often have poor oral hygiene. Weimers et al. used the OHAT and the Mann Assessment of Swallowing Ability (MASA), a screening test for dysphagia, to assess patients with a first stroke admitted to a rehabilitation hospital and reported that those with dysphagia had poor oral hygiene [13]. The MASA also assesses consciousness, cooperative behavior, auditory comprehension, aphasia (generalized speech impairment), dysarthria, and respiratory function [14]. In a study of hospitalized patients with acute heart failure, those with a poor OHAT not only had poorer swallowing function but were also older and had reduced skeletal muscle mass, nutritional status, and cognitive levels compared to those with a good OHAT [15]. In the present study, we found a correlation with OHAT in patients who were hospitalized for conditions other than pneumonia, because under normal conditions the oral environment is well maintained by thorough oral care during hospitalization, but deterioration of the patients’ general condition due to pyelonephritis, cerebral infarction, or other causes can worsen the OHAT score, and elderly nursing home residents have poor original swallowing function, which may explain the correlation between the OHAT and VF scores. This suggests that the OHAT is influenced by other factors, such as the patients’ general condition rather than the swallowing function itself.

In a study by Ekberg and Feinberg, age-related decline in swallowing function was also seen to be multisite, with 63% of the older patients (mean age 83 years) having oral abnormalities (difficulty ingesting, controlling, and supplying bolus to initiate swallowing) and 25% having pharyngeal dysfunction (bolus retention and tongue propulsion or paralysis of the pharyngeal contractile muscles); of the latter, 39% had abnormalities of the pharyngo-oesophageal segment (mostly dysfunction of the cricopharyngeal muscles) and 36% had oesophageal abnormalities [16]. When evaluating swallowing function in the institutionalized elderly, a contrast-enhanced swallowing evaluation that allows observation of all stages of swallowing is desirable.

The present study has some limitations. There was no set protocol for the need to assess swallowing in inpatients, and it was largely subjective to the facility staff. In addition, due to the wide range of background diseases in hospitalized patients and the varying severity of underlying diseases, disease-specific and pneumonia severity-specific assessments were not possible. It was not possible to mention disease-specific characteristics that affect swallowing function, such as the degree of pneumonia or dementia. Furthermore, oesophageal dysphagia, such as dyspnoea of passage, is difficult to evaluate with only 10 mL liquid contrast, which is an issue for future study.

## Conclusions

The relationship between the oral environment and swallowing function of nursing home residents was investigated. In facilities providing good oral care, the development of pneumonia may be related to factors other than the oral environment, and the OHAT score may reflect conditions other than swallowing function. Swallowing function in nursing home residents should be evaluated by a contrast-enhanced swallowing examination, which allows observation of all stages of swallowing.
